# Hygiene Efficacy of Short Cycles in Domestic Dishwashers

**DOI:** 10.3390/microorganisms13071542

**Published:** 2025-06-30

**Authors:** Matthias Kudla, Thomas J. Tewes, Emma Gibbin-Lameira, Laurence Harcq, Dirk P. Bockmühl

**Affiliations:** 1 Faculty of Life Sciences, Rhine-Waal University of Applied Sciences, 47533 Kleve, Germany; matthias.kudla@hochschule-rhein-waal.de (M.K.);; 2 NV Procter & Gamble Services Company SA, 1853 Strombeek-Bever, Belgium; gibbinlameira.e@pg.com (E.G.-L.);

**Keywords:** dishwasher, short cycle, hygiene, household, detergent, foodborne pathogens

## Abstract

This study investigated how factors associated with Sinner’s principle—namely temperature, time, and the chemical composition of detergents—affected the antimicrobial efficacy of domestic dishwashers, particularly during short cycles. These are of particular interest, because many consumers refrain from using long cycles while it is still unclear if short cycles can provide a sufficient level of hygiene. Thus, we chose a range of bacterial strains, including standard test strains such as *Micrococcus luteus* and *Enterococcus faecium*, as well as important foodborne pathogens such as *Escherichia coli*, *Staphylococcus aureus*, and Salmonella enterica. To account for the complexity of dishwasher cycles, we correlated hygiene efficacy with area under the curve (AUC) measurements derived from the respective cycle profiles. Our findings revealed that the reductions in *M. luteus* and *E. faecium* were minimally affected by the reference detergent. In contrast, a high-tier market detergent demonstrated a significant decrease in bacterial counts. Notably, both strains exhibited reduced efficacy at a main cycle temperature of 45 °C, suggesting that temperatures below 50 °C might represent a critical threshold at which the hygiene efficacy of domestic dishwashing processes declines. However, since food-related pathogens were more susceptible to the dishwashing process, even lower temperatures might deliver a sufficient level of hygiene. Plotting the logarithmic reduction/AUC ratio against the AUC indicated that the main cycle contributed approximately 10-fold more to microbial reduction than the rinse cycle. Furthermore, the antimicrobial impact of detergents was greatest at the lowest AUC values (i.e., during short cycles). Taken together, our results suggest that the applied chemistry may help to enhance antimicrobial performance especially in short dishwashing cycles.

## 1. Introduction

Domestic dishwashers have become indispensable appliances in many households, enabling more efficient cleaning of dishes compared to hand dishwashing while consuming fewer resources such as energy and water [1]. Although dishwashers may harbor numerous microorganisms [2], automatic dishwashing has been shown to provide a safe means to reduce pathogens on contaminated dishes, thus contributing to the control of foodborne diseases [3]. However, the constant decrease in cleaning temperatures to achieve a better energy efficiency might hamper the antimicrobial efficacy and consumer behaviors such as improper loading and insufficient cleaning and sanitization of the dishwasher might contribute to the presence and persistence of contaminants, although these effects still lack proper scientific evidence [4].

Despite the presence of different microbial colonizers, microbial contamination of dishes after dishwashing is generally assumed to be very low [2]. Nevertheless, research suggests that lower temperatures (i.e., below 50 °C) can lead to higher bacterial diversity and potentially insufficient microbial reduction [3]. Both cleaning and antimicrobial effectiveness depend on the interplay of mechanical action, temperature, detergent composition, and cycle duration [3,5]. Recent studies have specifically examined the impact of dishwashing detergents on cleaning efficacy, especially in shorter cycles, supporting this concept. As predicted by Sinner’s principle, it has been shown that a decrease in process time can be compensated by better detergent chemistry at a constant temperature, leading to a comparable cleaning efficacy [6]. However, it remains unclear whether this effect can also be achieved in terms of the reduction in and removal of microorganisms. Similar to certain stains (e.g., tea) that are not removed but rather bleached, a reduction in microbial cells on a dish surface can be achieved by inactivating the cells rather than removing them by physico-chemical means. Since this combination of removal and inactivation is likely driven by all four factors of Sinner’s principle, it is worthwhile considering the role of each factor, especially at lower temperatures. Although systematic investigations have been conducted previously, data on the behavior of market detergents, especially with regard to shorter cycles, are lacking. This gap is of particular importance, as automatic dishwashing plays a crucial role in controlling the risk of infection in the domestic environment, ensuring the removal of pathogens after handling high-risk foods, such as raw poultry, thereby breaking potential chains of infection [7]. The emphasis on lower energy consumption in the development of household appliances has led to a situation where relying on temperature alone to achieve an adequate reduction in microbial cells on contaminated dishes is insufficient. Unlike sterilization or most medical device reprocessing procedures, temperature alone cannot guarantee effectiveness, making simpler mathematical models, such as the A0 concept [8], unsuitable for describing the complex interactives of dishwasher cycle profiles (which include various heating phases and periods of constant temperatures during the main wash and rinse cycles). This complexity calls for more sophisticated approaches. Interestingly, initial attempts to link the combined effects of time, temperature, and chemistry on microbial reduction have already been made in the context of domestic dishwashing and laundering, which might serve as a base for further concepts to be developed [9,10].

In the current study, we use a freely programmable standard household dishwasher to investigate the hygiene efficacy of cycles with different durations at temperatures representative of the lower end of current market offerings. By using a high-tier market detergent, the standard detergent for normative testing of dishwasher efficacy, and water alone, we aim to develop a comprehensive understanding of the hygiene efficacy of automated dishwashing processes, particularly in relation to shorter cycles and lower temperatures. In addition to providing new systematic data on microbial reduction in domestic dishwashing with regard to time, temperature, and chemistry, this study seeks to delineate the hygienic boundaries of domestic dishwashing, which has traditionally been focused on energy-saving considerations. By introducing a mathematical model to link the antimicrobial efficacy of both low- and high-performing automatic dishwashing detergents to the cycle profiles of the dishwasher, we also aim to contribute to a general understanding of the interplay between all factors of Sinner’s principle, thereby paving the way for future efforts to balance hygiene and sustainability.

## 2. Materials and Methods

### 2.1. Dishwasher and Cycle Test Parameters

A freely programmable standard household dishwasher (85 cm height, 60 cm depth, 60 cm width) based on Bosch E-Nr.: SMS2ITW33E/34 (Robert Bosch Hausgeräte GmbH, Munich, Germany) was used to investigate the cleaning efficacy of various low-consumption time–temperature profiles. A top-tier market detergent in pouch-form was tested alongside a reference detergent to evaluate their hygiene performance under different test conditions. An amount of 50 g of ballast was added to each run, in accordance with the IKW protocol [11].

The total durations of the test cycles were set at 30 min, 40 min, 90 min, and 180 min. A range of main wash temperatures were used, ranging from 30 °C to 70 °C, with water volumes of either 8 or 11 L. The water volume was measured via a water meter attached to the pipe and additionally compared with the integrated program of the household dishwasher. Rinse cycles were mostly performed at 50 °C, although 40 °C was used in two of the experiments. Table 1 lists the cycle parameters for all the programs tested.

In addition to the programmed short cycles, a 90 min cycle and an average eco-cycle according to [12] were included (code VII and code VIII, respectively). The water consumption of both cycles was 10.95 L, comprising 4.07 L in the main wash, 3.34 L in the first rinse, and 3.54 L in the final rinse. The cycle lengths were either 90 or 180 min, with the main wash and final rinse temperatures both set at 50 °C.

### 2.2. Dishwasher Program Sequence

**Figure 1:**
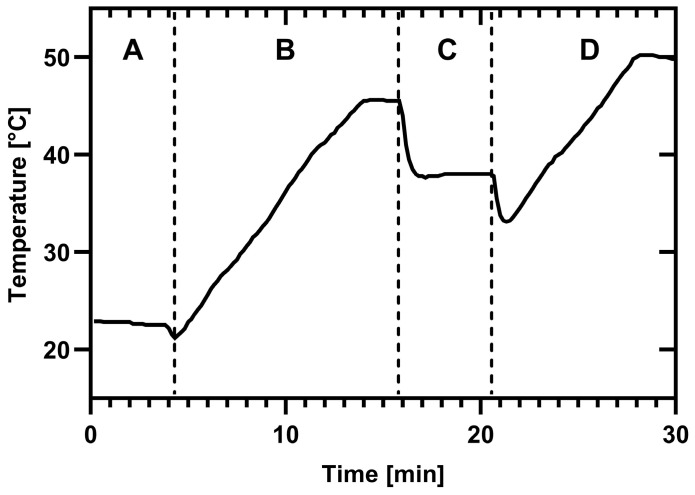
The typical sequence of each cycle, highlighting the distinct phases: phase A—initial draining combined with the influx of freshwater; phase B—simultaneous heating of the machine and activation of the spray arms with detergent, followed by the drainage of heated water; phase C—supply of fresh soft water without heating or detergent, ending with the drainage of remaining water; and phase D—influx of new water with the addition of rinse aid, if necessary, accompanied by the operation of the spray arms and final drainage of the remaining water inside the dishwasher.

After pressing the main button on the programmable dishwasher, all cycles were initiated. An initial drain phase begins, even though no water residues are present in the sink. This drain phase stops automatically once the internal sensor detects there is no water flow in the drainage system, and the machine subsequently fills with freshwater (Figure 1; phase “A”). The main cycle commences with the opening of the dispenser, releasing the detergent. After, the heating phase starts simultaneously with the activation of the spray arms for the main wash, with the pump operating at 3500 revolutions per minute (rpm). The spray arms work in an alternating sequence. The lower spray arm is used first, followed by the upper spray arm, each for an equal duration, and this process is repeated. Once the destined temperature is reached, the heating element switches off, and the cleaning continues. At the end of the main wash, the water is drained again (main wash illustrated as phase “B” in Figure 1). A first rinse process is initiated by the influx of freshwater without heating. Again, the lower spray arm operates first, followed by the upper spray arm. This process ends with another drainage of water (phase “C” in Figure 1). Following the influx of freshwater in the final rinse phase (phase “D” in Figure 1), rinse aid is added, either manually or automatically. In the case of the reference detergent, rinse aid was added manually. No rinse aid was added with the market detergent. Again, both spray arms operate alternately, starting with the lower spray arm. Each spray arm runs twice while the device simultaneously heats the water to reach the final rinse temperature. The heating element was turned off after the designated temperature was reached and a final drain phase occurs after the rinse phase was completed. The cycle concludes after the completion of the final rinse phase.

### 2.3. Dishwashing Detergents

For the examinations, either a top-tier market detergent in capsule form (Procter & Gamble Brussels, Belgium; pH: 10.7) or reference detergent E (Wfk Testgewebe GmbH, Brüggen, Germany; pH: 10.8) with reference rinse aid C (Wfk Testgewebe GmbH, Brüggen, Germany) was used (Tables S1 and S2). The quantity of the reference detergent used was 14.5 g, with 2 mL of rinse aid added manually using a pipette by briefly opening the machine at the appropriate time (i.e., in the middle of the rinse aid step, marked as phase “D” in Figure 1).

### 2.4. Preparation of Ballast Soil

The 50 g ballast soil units were prepared according to the instructions provided in IKW Recommendations for the Quality Assessment of the Cleaning Performance of Dishwasher Detergents [11].

### 2.5. Preparation of Bio-Monitors

Stainless austenitic bio-monitors were prepared as per standard IEC 63331 [13]. All bio-monitors were cleaned in an ultrasonic bath (USC-900TH, Avantor Inc., Darmstadt, Germany), air-dried, and sterilized in an autoclave (VX-65, Systec GmbH & Co. KG, Linden, Germany). After, autoclaved bio-monitors were coated in a bovine albumen, mucin, and cornstarch (BAMS) soil matrix, containing a specific bacterial strain.

### 2.6. Preparation of BAMS Soil Matrix

The BAMS soil matrix was prepared according to standard IEC 63331; 0.3 g mucin (91053-71-3, Carl Roth GmbH + Co.KG, Karlsruhe, Germany) and 0.18 g of bovine serum albumin fraction V (BSA, 9048-46-8, Carl Roth GmbH + Co.KG, Karlsruhe, Germany) were dissolved in 20 mL of sterile water and heated to 50–60 °C under continuous stirring (magnetic stirrer RSM-04H, Phoenix Instrument, Garbsen, Germany). A second solution consisting of 0.9 g maize starch (9005-25-8, Carl Roth GmbH + Co.KG, Karlsruhe, Germany) was dissolved in 2 mL water and another 8 mL of sterile water was heated until boiling point. The dissolved corn starch was directly transferred to the boiling water, which resulted in the thickening of the liquid. When both solutions reached room temperature, they were mixed, resulting in a total volume of 30 mL of BAMS.

### 2.7. Antimicrobial Performance

Before initiating the tests, an intensive program was implemented to ensure optimal starting conditions. Experiments were carried out in accordance with the standard method based on DIN 10512, using *Enterococcus faecium* (DSM 2146), *Micrococcus luteus* (DSM 1790), *Staphylococcus aureus* (DSM 794), *Escherichia coli* (DSM 682), and *Salmonella enterica* subsp. enterica (DSM 5569) to assess microbiological hygiene efficacy. While *Enterococcus faecium* and *Micrococcus luteus* were chosen as standard bacterial strains, the strains *Staphylococcus aureus*, *Escherichia coli*, and *Salmonella enterica* were chosen as typical kitchen-related food pathogens. As these standard tests are not designed for other microbial contaminants (fungal strains and viruses), only different bacterial strains were used for these tests. Moreover, unlike bacterial pathogens, fungi are not supposed to play a crucial role in the transmission of foodborne infections [2]. Prior to use, bacterial strains were suspended in an 80% glycerol solution at −80 °C. They were thawed at room temperature and then plated on Tryptic Soy Agar (TSA, 105458, Merck Chemicals GmbH, Darmstadt, Germany) using a sterile inoculation loop. From the surface culture on TSA, second and third subcultures were prepared by transferring individual bacterial colonies to new agar plates. Three TSA plates containing the last bacterial subculture were each rinsed with 10 mL of sterile 0.9% NaCl solution. The suspension was centrifuged for 10 min at 4696 g (Multifuge X3R, Thermo Fisher Scientific, Wesel, Germany). The supernatant was then discarded, and the washing step was repeated once. After washing, the pellet was resuspended in 10 mL of BAMS.

Three austenitic steel bio-monitors were contaminated with 100 µl BAMS containing either *Enterococcus faecium*, *Micrococcus luteus*, *Staphylococcus aureus*, *Escherichia coli*, or *Salmonella enterica* for every run. The test contamination was applied to the longitudinal grain side of each bio-monitor and evenly distributed. After a drying time of 4 h at 22 °C and 70% relative humidity, the number of colony-forming units (CFUs) per bio-monitor was determined. The bio-monitors were clamped in modified holders parallel to the plate surface and the plates containing the bio-monitors were placed in the corresponding empty holders of the dishwasher. The machine was fully loaded with dishes (13 place settings), according to IEC 60436. For every run, 50 g of ballast was added inside a mug in the top rack of the dishwasher, following IKW recommendations [11].

After the run, any bacteria that remained embedded in the matrix of the bio-monitors were extracted using extraction liquid (EL). One liter of EL consists of 30 g Tryptic Soy Broth (TSB, 41111700, Merck Chemicals GmbH, Darmstadt, Germany), 30 g L^−1^ Tween 80 (9005-65-6, AppliChem GmbH, Darmstadt, Germany), 0,3 g L^−1^ lecithin (36486, Alfa Aesar, Thermo Fisher Scientific, Wesel, Germany), 1 g L^−1^ histidine (200-745-3, Carl Roth GmbH + Co.KG, Karlsruhe, Germany), and 5 g L^−1^ sodium thiosulfate (231-867-5, AppliChem GmbH, Darmstadt, Germany). The EL itself not only extracted the bacteria from the matrix, but also negated the effects of possible detergent residues that might interfere with the bacterial count. An amount of 5 mL of EL was transferred into test tubes with screw caps together with the bio-monitors to initiate the extraction via vortexing the tubes for 3 s (VV3, Avantor Inc., Darmstadt, Germany). Subsequently, the tubes were placed on a roller mixer (ROLLER 10 digital, IKA-Werke GmbH & Co. KG, Staufen, Germany) for 10 min at 80 rpm. Depending on the expected number of bacteria left in the EL, around 100–500 µL of the EL was transferred to corresponding TSA plates and incubated at either 30 °C for 48–72 h for *M. luteus* (HPP110, Memmert GmbH & Co. KG, Schwabach, Germany) or 37 °C for 24–48 h for all other bacterial strains. Finally, all TSA plates were checked for the remaining number of bacteria and the logarithmic reduction (LR) factor was determined using the formula below. Each condition was performed in triplicates.

$$LR={log}_{10}\left(initial\;microbial\;load\right)-{log}_{10}(remaining\;microbial\;load)$$

### 2.8. Execution of the Dishwasher Test Runs

Prior to testing, the programmable dishwasher was cleaned by running an intensive cycle profile (70 °C main wash temperature). Subsequently, the filter, spray arms, and the sump were checked. All experiments were performed in triplicate. The experiments were performed alternately with reference detergent E/rinse aid, market detergent, and only water. After every four to six runs, an intensive cycle using a market detergent was performed to counteract cumulative soiling effects and/or possible bacterial cross-contamination inside the dishwasher machine. Immediately before starting a test run, a cold rinse program was started to set the initial temperature to approximately 18 to 23 °C. After every 100–200 L of water consumption, a regeneration cycle was performed (Somat Salz, Henkel AG & Co KGaA, Düsseldorf, Germany). Water hardness was monitored after the regeneration cycles and several experiments (Testbesteck VISOCOLOR^®^ ECO Gesamthärte, Macherey-Nagel GmbH & Co. KG, Düren, Germany); a constant value between 1 and 2°dH was maintained throughout.

An amount of 50 g frozen ballast soil was placed underneath a mug in the upper rack and the cleaner was placed in the dispenser. After fully loading each machine, the test programs were started and run to completion. The cleaner was put in the dispenser. After the machines were fully loaded, the test programs were started. A temperature logger TELID311 RFID (Microsens GmbH & Co. KG, Erfurt, Germany) was placed on top of the cutlery basket, and with a measurement interval of 10 s, the temperature was recorded (Figure S1).

### 2.9. Statistical Analysis and Mathematical Approach

Logarithmic reduction (LR) values were analyzed using one-way ANOVA, with statistically significant differences determined by Tukey’s multiple comparison test, which identifies significant differences among the mean values of non-normally distributed data within the observed groups.

**Figure 2:**
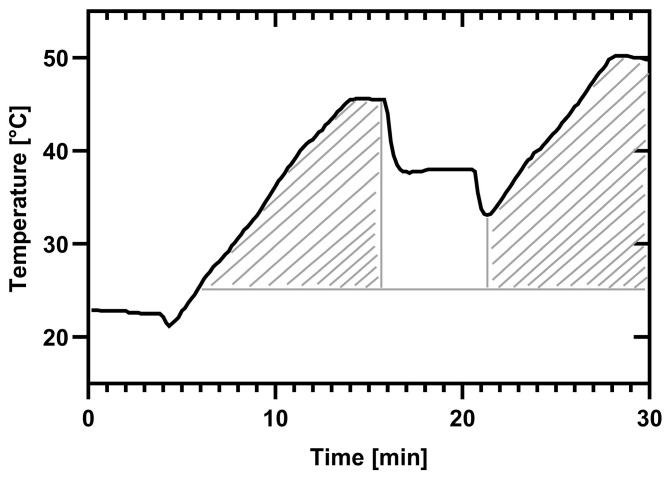
Areas under the curves (AUC) illustrated for an exemplary program. All temperatures ≥ 25 °C (highlighted area) in the main wash and in the final rinse were considered.

The A0 concept typically expresses the relationship between the temperature applied and the time of exposure required to achieve a defined inactivation of microorganisms in disinfection procedures [8]. In this context, however, the A_0_ concept is defined as a limiting value at which the dishwasher achieves maximum microbial hygiene efficacy. A mathematical approach was implemented to assess the influence of temperature, time, chemical reactions, and the LR of bacteria. To quantify the combined effects of temperature and duration, including heating phases, the area under the curve (AUC) was calculated for each cycle code (Figure 2). For the AUC, temperature (x) was related to time (t) using the following formula:

$$AUC=\sum_{i=1}^{n-1}\frac{\left(x_{\left(i+1\right)}+x_{i}\right)*(t_{\left(i+1\right)}-t_{i})}{2}$$

Here, the AUC of the main cycle (phase “B” in Figure 1) and the AUC of the final rinse cycle (phase “D” in Figure 1) were calculated separately. All quantifications are summarized in Table 2 The combined value for temperature and duration of the programs, including heating phases, was calculated for each code (Figure 2 and Table 2).

## 3. Results

### 3.1. Assessment of Antimicrobial Efficacy of Different Dishwashing Programs

To investigate the hygienic reprocessing of contaminated dishes in domestic dishwashers, tests were conducted according to IEC 63331 [13] in a freely programmable dishwasher using two different detergents and water. The detergents included the IEC standard detergent and a top-tier market detergent in pouch-form, representing low- and high-performance products, respectively. The focus on short cycles at lower temperatures aimed to identify potential hygiene boundaries (i.e., the point at which the efficacy of inactivating microbial cells or removing them from surfaces is reduced), while also considering the impact of the performance level of the used detergent. As required by standard IEC 63331, *Micrococcus luteus* was the primary test strain, with additional bacterial species (i.e., *Enterococcus faecium*, *Escherichia coli*, *Staphylococcus aureus*, and *Salmonella enterica*) included to represent important food-related pathogens.

*Micrococcus luteus*, being the test strain indicated in IEC 63331, was investigated across the largest range of cycles. This included durations of 30 and 40 min, with main wash temperatures of 30–60 °C and rinse temperatures of 40–50 °C, respectively (codes I–VI and IX–X; Table 1). In addition, two separate cycles resembling a typical eco-cycle and a “shortened” eco-cycle were tested, featuring a duration of 90 and 180 min (code VIII and code VII, Table 1). Both codes had a main wash and rinse temperature of 50 °C and their water consumption was higher (11 L) compared to the other cycles (8 L).

**Figure 3:**
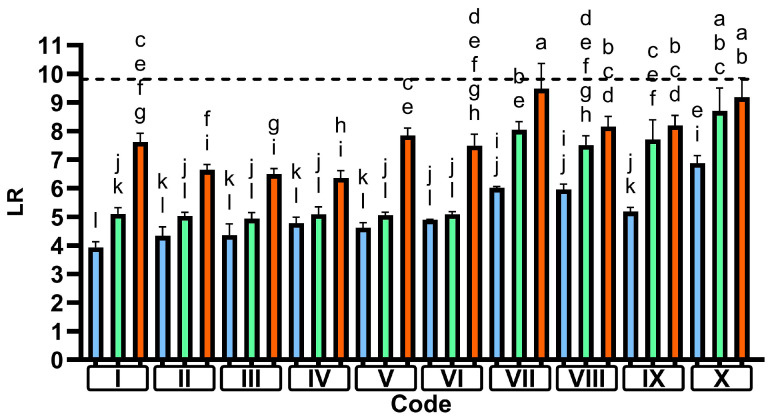
Reduction in *Micrococcus luteus* in different programs with water (blue), IEC standard detergent (green), and market detergent (red). Parameter combinations for the indicated program codes are described in Table 1. The dotted line represents the maximum possible reduction relative to the initial cell count. The letters above each bar indicate a different significance level through a statistical analysis of one-sided ANOVA with Tukey’s multiple comparison test. Groups with the same letter are not statistically different, while groups with different letters have statistically different means.

In all tested cycles, the achieved reduction was lowest when no detergent was used and highest with the market detergent (Figure 3). Interestingly, in short cycles with a main wash temperature up to 50 °C (codes II–VI), the antimicrobial performance of the IEC standard detergent was not significantly better than water, while in higher-temperature cycles (60–70 °C; codes VII–X), IEC-E matched the market detergent and outperformed water. Only code I, with a main wash temperature of 30 °C, allowed for statistical differentiation between water, IEC-E, and the market detergent, with efficacy increasing in this order.

The other bacterial strains were tested in three cycles: codes IV and VI, which correspond to the short cycles, and code VIII, which resembles an eco-cycle. All cycles had a rinse temperature of 50 °C. This set of experiments aimed to address whether short cycles might pose a risk of infection; therefore, both Gram-negative and Gram-positive foodborne bacteria were chosen to cover a range of relevant potential pathogens. Previous studies suggest that temperatures around 50 °C might be a crucial point of differentiation [12], and, to the best of our knowledge, there are few, if any, short cycles present on the market with cleaning temperatures below 45 °C [12].

**Figure 4:**
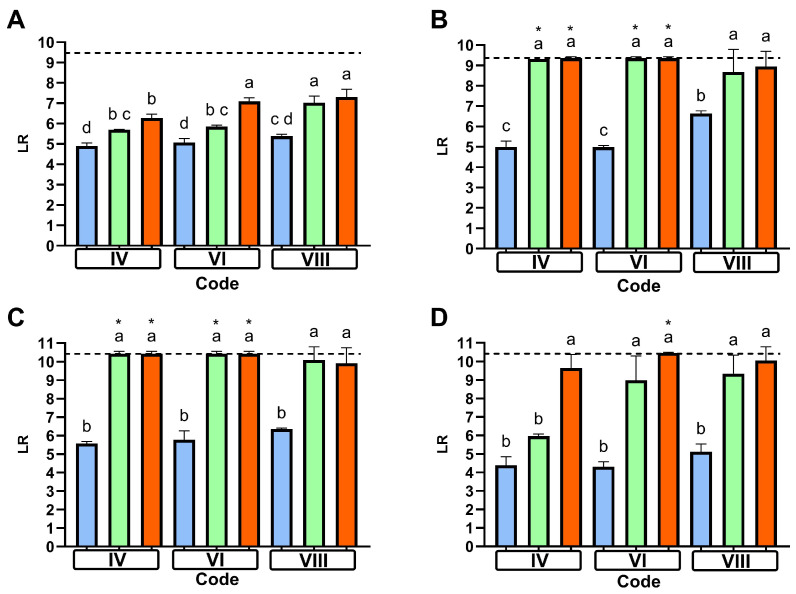
Reduction in *Enterococcus faecium* (**A**), *Escherichia coli* (**B**), *Salmonella enterica* (**C**), and *Staphylococcus aureus* (**D**) across selected programs with water (blue), IEC standard detergent (green), and market detergent (red). Parameter combinations for the indicated program codes are described in Table 1. The dotted line represents the maximum possible reduction relative to the initial cell count. The letters above each bar indicate a different significance level through a statistical analysis of one-sided ANOVA with Tukey’s multiple comparison test. Groups with the same letter are not statistically different, while groups with different letters have statistically different means. The stars above each letter indicate that bacteria were completely absent on the bio-monitors.

*Enterococcus faecium*, typically chosen as a temperature-resistant test strain [14], showed a similar reduction across all cycles tested without detergent. However, with the IEC standard detergent, the reduction was lower in cycle codes IV and VI, than in code VIII. In the latter cycle, the efficacy of both detergents was comparable. For this test strain, the market detergent, while still being more effective than the IEC standard detergent, showed a significant drop between 50 °C and 45 °C (Figure 4A).

Notably, there was no significant difference between the two detergents across all tested codes for *E. coli*. However, the reduction was clearly greater with the addition of detergent compared to water alone (Figure 4B). In general, *S. enterica* behaved in a very similar way to *E. coli* (Figure 4C).

For *S. aureus*, both detergents were more effective than water in all tested codes, although for the IEC standard detergent, the effect was not significant in code IV. The hygiene efficacy of the IEC standard detergent decreased substantially with a lowered main wash temperature, while the market detergent exhibited a slight, though not significant, reduction in the short cycles (Figure 4D).

It should be noted that for all tested bacteria, except for *M. luteus* and *E. faecium*, the reduction achieved with detergents in the eco-cycle (code VIII) was generally lower than in the shorter cycle using the same main and rinse temperatures (code VI). Although this reduction was not statistically significant, the consistent observation of this trend suggests a minor, yet real phenomenon.

### 3.2. Mathematical Approach to Determine the Hygiene Efficacy of Dishwashing Processes

**Figure 5:**
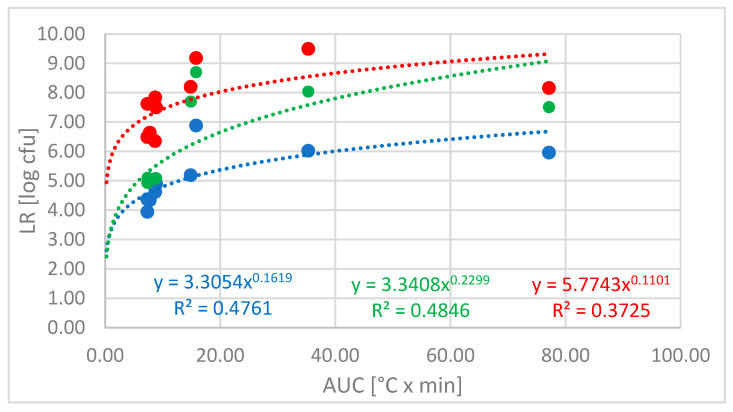
Log reduction for *Micrococcus luteus* across the investigated codes with water (blue), IEC standard detergent (green), and market detergent (red) in relation to the AUC. Dotted lines indicate a possible mathematical dependency, including the corresponding formula and determination coefficients.

The complexity of dishwasher cycles, featuring variable and constant temperature phases during washing and rinsing, makes it challenging to explain how the program parameters affect hygiene efficacy. Moreover, the antimicrobial effects of the added detergents may involve different modes of action, such as killing or inactivation (e.g., through oxygen bleach) and physical removal of cells (by detergency action). To better understand the relationship between the achieved microbial reduction from specific cycle/detergent combinations and the underlying cycle parameters (mainly determined by time and temperature), the LR for *M. luteus* was plotted against the AUC calculated for each code (Figure 5).

The trend lines were fitted to an exponential function with a detergent-dependent factor, using the AUC value as the base for a variable exponent. This approach reveals a possible relationship between the AUC and the resulting LR, in which the market detergent appears to exert a greater influence on the LR at low AUC levels, while the IEC standard detergent demonstrates a similar LR to water at these low AUC levels. However, at AUC values greater than 10, this trend appears to change, revealing a performance gap between water and the detergents.

**Figure 6:**
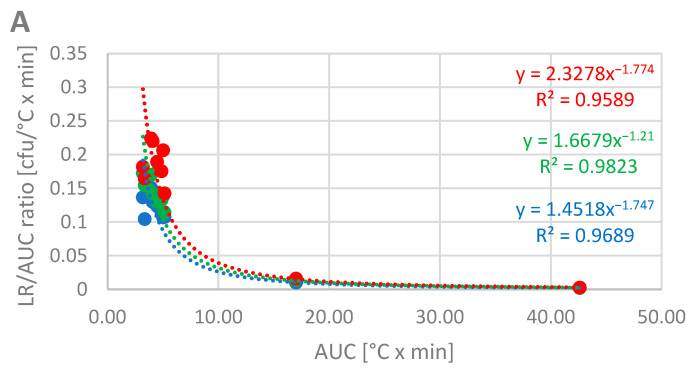

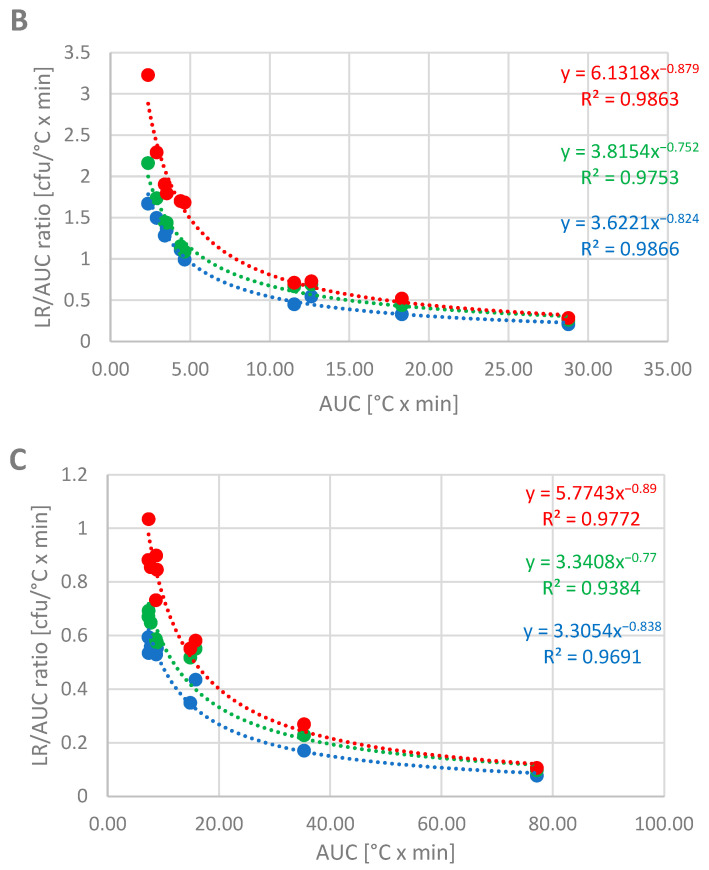
LR/AUC ratio for *Micrococcus luteus* across the investigated codes with water (blue), IEC standard detergent (green), and market detergent (red) in relation to the AUC. The LR/AUC ratio was calculated for the AUC in the rinse phase (**A**), the main wash (**B**), or the total cycle (**C**) and plotted against the corresponding AUC.

Plotting the LR/AUC ratio against the AUC reveals additional correlations between the detergent-dependent antimicrobial effect and the program structure (Figure 6). The calculated trend lines, which represent a similar exponential function as used in Figure 5, show a less pronounced spread when plotted against the AUC of the rinse phase (Figure 6A) compared to the AUC of the main wash (Figure 6B). Moreover, although the AUC levels for both cycle phases are comparable, the LR/AUC ratio per AUC is approximately ten-fold higher for the main wash than for the rinse. Consequently, the LR/AUC ratio is positioned at an intermediate level when plotted against the total AUC (Figure 6C).

## 4. Discussion

The antimicrobial performance of an automatic dishwashing process is crucial for reducing the risk of foodborne infections in domestic settings, as hand dishwashing often fails to deliver significant reductions in foodborne pathogens on dishes and other food-contact surfaces [15]. Although studies conducted in consumers’ homes suggest that the level of contamination found on cleaned items after dishwashing is very low [2], it has been shown that the microbial reduction achievable in a domestic dishwasher depends on several factors, including technical parameters like cycle duration and temperatures in both the main wash and rinse phases, as well as the choice of detergents [3,14]. In this context, the impact of activated oxygen bleach has been investigated [14], while the impact of detergent performance per se remains unclear. It must, however, be assumed that cleaning performance plays a major role in determining the antimicrobial efficacy of dishwashing, since the physical removal of microbial cells, even if they are not inactivated or killed, will contribute to the decontamination of hard surfaces [5].

Sinner’s principle [16] applies to automatic dishwashing as it does to any other cleaning processes, indicating that the roles of time and temperature must be considered alongside the chemical effects of detergents. Mechanical action can be considered as a constant factor due to the similar principles that are applied in virtually all domestic dishwashers. Like in other domestic appliances, the strive for lower energy consumption has led to a regulatory framework that strongly favors long cycles at relatively low temperatures, referred to as “eco” cycles [1]. However, these longer cycles are not always accepted by consumers due to their duration [17]. Consequently, discussions have emerged regarding the suitability of short cycles, as alternative sustainable programs, which have shorter durations while maintaining low energy consumption levels [18]. According to Sinner’s principle, a decrease in time and/or temperature can be compensated by the increased use of chemistry or mechanics; however, adjustments to the latter are limited since the operational mechanics of a dishwasher cannot be easily changed. Tewes et al. demonstrated that market detergents could achieve cleaning performance in some short cycles that is comparable to that of longer “eco” cycles [6]. Unfortunately, a study on laundering showed that Sinner’s principle has some limitations in terms of its adaptability to the antimicrobial efficacy of cleaning processes, mainly because temperature is a crucial factor for inactivating microbial cells [19]. Therefore, this study investigated the hygiene efficacy of automatic dishwashing processes for domestic applications, focusing on potential boundaries of short cycles and the impact of different detergent formulations. Since the chemical compositions of market detergents are not available to the public, it was not possible to comprehensively analyze the impact of specific ingredients.

In general, our findings suggest a good hygiene efficacy for the investigated dishwashing processes with an LR of >5 of both tested detergents, even at low temperatures in very short cycles. This might be, at least partly, explained by the fact that the dishwashing process can be assumed to be effective at removing the matrix of bovine albumin, mucin, and cornstarch (according to IEC 63331 [13]) from hard surfaces, thereby also removing most of the microbial cells embedded in this matrix [6]. However, our results also show that a considerable number of cells remain on the surfaces, necessitating either more stringent removal methods or inactivation. This can be achieved through the use of a more potent detergent, which can deliver up to a 2.5 log higher reduction under the same cycle conditions, or by increasing the cycle time and/or temperature (Figure 3). This correlation network is clearly visualized in Figure 5, where the hygiene performance of the standard detergent resembles that of water at AUCs of <10. At higher AUCs, the differences are less pronounced, suggesting a bigger impact of time and/or temperature. Moreover, this figure suggests that while the use of a detergent enhances the hygiene performance across all cycle profiles, the antimicrobial impact of a high-performing formulation is more pronounced at low AUCs (i.e., in shorter cycles). Using the AUC as the independent variable appears to be more suitable than simply comparing the LR to the maximum temperature reached in the main wash and rinse, as differentiation is not well represented for cycles between 45 °C and 50 °C (Code IV and VI). Nonetheless, Figure S2 clearly indicates high antimicrobial efficacy in cycles with a maximum temperature (T_max_) of ≥60 °C, regardless of the detergent used, and decreased hygiene performance in the cycle with the lowest main wash temperature (30 °C), although this effect is less pronounced for the market detergent. While the AUC is a concept that has been successfully applied to calculate the antimicrobial efficacy of heat-based disinfection in medical devices (A_0_ concept; [8]), our data suggest a more complex interaction among time, temperature, and chemical factors that collectively influence the hygiene efficacy of dishwashing processes. In this context, the detergent appears to have a greater impact on antimicrobial efficacy during the main wash compared to the rinse phase, as illustrated in Figure 6. Plotting the LR/AUC ratio against the AUCs of the total cycle, the main wash, and the rinse reveals that the LR of different detergents or water varies with AUCs in the main wash but not in the rinse. This phenomenon can be explained by the absence of detergent in the rinse. Furthermore, this analysis shows that the additional impact of an AUC increase in the rinse on the antimicrobial efficacy is less significant than that of an AUC increase in the main wash, especially when a market detergent is used.

As specified in the standard IEC 63331, the efficacy tests focused on *Micrococcus luteus*, a strain known for its differential response to various antimicrobial impacts, thus representing a “worst case scenario” [13,14]. However, it is important to understand how other microorganisms, particularly kitchen-relevant strains, react in the dishwasher in order to assess potential infection risks due to insufficiently decontaminated dishes. So, we tested a range of strains representing both Gram-positive and Gram-negative bacteria, as well as important food pathogens.

Only selected cycles were tested using these strains, including a typical eco-cycle (code VIII) and two short cycles (codes IV and VI), one of which had a lower main wash temperature (code IV) than the other. Main wash temperatures of 45 °C and 50 °C were chosen based on our data (Figure 3) and findings from a recent study by Brands et al. [14], which suggest these temperatures might represent a “critical threshold” where microbial reduction in domestic dishwashing could decline.

A drop in antimicrobial performance of the market detergent between 50 °C (code VI) and 45 °C (code IV) was observed in tests using *E. faecium*. In contrast, this effect was not seen with *S. aureus* or *S. enterica*, although the log reduction of the standard detergent significantly decreased when temperature was lowered from 50 °C to 45 °C. In this regard, the tests with *M. luteus* suggest that the temperature-related performance drop with the standard detergent might occur already between 60 °C and 50 °C (Figure 3, codes VI and VIII). For *E. coli*, a slight (but not significant) decrease in antimicrobial performance could be observed between 50 °C and 45 °C for the standard detergent. This is in line with the findings of Brands et al., who observed similar effects with *M. luteus* and *E. faecium* when using an IEC standard detergent [14].

Overall, it can be concluded that the main wash temperature has a considerable effect on the antimicrobial performance of an automatic dishwashing process, depending on the performance level of the detergent and the investigated test strain. Data suggest that the critical temperature for short cycles may be below 60 °C or 50 °C when using low- or high-performance detergents, respectively. The antimicrobial impact of a detergent is highest at low AUC values, indicating that shorter cycles require an additional chemical component to achieve better hygiene efficacy. Interestingly, only market detergents seem capable of compensating for the lack of antimicrobial efficacy in short cycles, while in longer cycles, even low-performing products can enhance hygiene efficacy. This discrepancy might be attributed to the varying cleaning efficacy of the detergents, which has been shown to differ significantly in short cycles [6]. However, this hypothesis cannot be fully answered within the scope of this study and requires further investigation.

It is important to note that our findings regarding antimicrobial efficacy do not allow for definitive conclusions about potential infection risks, as more information would be needed to make such assessments. Likewise, it is not possible to define a specific log reduction that should be achieved during the dishwashing process. Developing such recommendations would require data on microbial species and counts on dishes, their infectious doses, and exposure scenarios. Nevertheless, when compared to other standardized methods that evaluate disinfection efficacy, such as those used in washing machines [20], which require a log reduction of >7, it can be inferred that short cycles in domestic dishwashers deliver sufficient antimicrobial efficacy at temperatures greater than or equal to 50 °C when using highly pathogenic strains such as *M. luteus* and *E. faecium*, whereas 45 °C is sufficient when more kitchen-relevant strains such as *E. coli*, *S. aureus*, and *S. enterica* are considered. This, however, must be considered a general recommendation that requires more comprehensive investigations into the impact of different detergent ingredients on antimicrobial performance. Likewise, long-term effects on microbial contamination in domestic dishwashers have not been considered, but might pose a problem, depending on the cycles used, thus requiring further research.

## 5. Conclusions

Short cycles in domestic dishwashers are effective in reducing the microbial load on contaminated dishes as LRs of >5 were observed in cycles with main wash and rinse temperatures around 50 °C, respectively, even in the absence of detergent. When using a market detergent, an LR of >7 can be achieved in programs with a main wash temperature of ≥50 °C. Interestingly, the range around 45 °C appears to be a critical temperature threshold for the standard germ *M. luteus*, as more germs survived the dishwasher cycle than in ranges >50 and <40 °C. The exact cause of this phenomenon could not be precisely determined in the course of these experiments, which is why the focus of a future study could be on determining this temperature threshold.

Overall, the presence of detergents increased hygiene efficiency compared to water alone. In some specific codes, the performance with the market detergent was similar to the reference detergent. This includes codes that had either main wash temperatures > 50 °C or a dishwashing cycle duration longer than 90 min. In contrast, all investigated foodborne pathogens showed no significant difference between the market and the reference detergent. Only *S. aureus* was able to persist at 45 °C in the presence of the reference detergent.

Considering the AUC of a cycle temperature profile, it is possible to roughly predict the LR that can be achieved in a domestic dishwasher when using only water or a different type of detergent. This predictive capability might serve as a versatile tool for the hygiene performance attainable with specific dishwashing cycles, or for setting limits for temperature profiles to ensure the safe reprocessing of contaminated dishes while optimizing energy efficiency.

## Figures and Tables

**Table 1:** Tested short-cycle parameter combinations.

Code	T main [°C]	Time [min]	Water [L]	T rinse [°C]
I	30	30	8	50
II	40	30	8	50
III	45	30	8	40
IV	45	30	8	50
V	50	30	8	40
VI	50	30	8	50
VII	50	90	11	50
VIII	50	180	11	50
IX	60	40	8	50
X	70	40	8	50

**Table 2:** Calculated AUC values [$\frac{{}^{\circ}C\;\times \;min}{100}$] of all codes considering temperatures ≥ 25 °C.

Code	I	II	III	IV	V	VI	VII	VIII	IX	X
AUC	7.37	7.77	7.36	8.68	8.73	8.87	35.31	77.13	14.88	15.81

## Data Availability

The original data presented in the study are openly available at https://opus4.kobv.de/opus4-rhein-waal/frontdoor/index/index/docId/2215, accessed on 30 June 2025.

## Supplementary Materials

The following supporting information can be downloaded at: https://www.mdpi.com/article/10.3390/microorganisms13071542/s1, Figure S1: temperature curves of all codes; Figure S2: log reduction for *Micrococcus luteus* in dependence on the maximum temperature in the investigated codes; Table S1: ingredients list of reference detergent E; Table S2: ingredients list of market detergent.

## Abbreviations

The following abbreviations are used in this manuscript:

AUC	Area under the curve
LR	Logarithmic reduction
