# Supplementary material for: Hygiene Efficacy of Short Cycles in Domestic Dishwashers
Source: Microorganisms. 2025 Jun 30;13(7):1542. doi: 10.3390/microorganisms13071542 (PMC12299907; doi:10.3390/microorganisms13071542)
Supplement: Supplementary file 1 [file microorganisms-13-01542-s001.zip › microorganisms-3684142-supplementary/microorganisms-3684142 supplementary material/Table S1 & Table S2 of microorganisms-3684142.pdf]

Supplements

Table S1: Ingredients list of reference detergent E

Chemical substance	Weight % (w/w)
Sodium citrate dihydrate	30..0
Maleic acid/acrylic acid copolymer sodium salt	6.0
Sodium percarbonate	7.0
Tetraacetyl ethylene diamine (TAED)	2.0
Sodium disilicate	1.5
Linear fatty alcohol ethoxylate 100%	2.0
Protease	1.0
Amylase	0,5
Sodium carbonate	Approx. 50%

Table S2: Ingredients list of top-tier market detergent in pouch-form.

Chemical substance
Trisodium salt of methylglycinediacetic acid
Sodium Carbonate Peroxide
Sodium Carbonate
Aqua
Trideceth-n
PPG/PEG/PPG-3/14.5/16 Propylheptyl Ether
Tetrasodium Etidronate
Sodium Sulfate
Disodium Disilicate
Dipropylene Glycol
TAED
Copolymer of acrylic and sulphonic acids
SODIUM ACRYLATE/SODIUM ACRYLOYLDIMETHYL TAURATE COPOLYMER
Protease
PARFUM
Polyvinyl alcohol<br>Titanium Dioxide
Disodium Phosphite
Cellulose Gum
Glycerin
zinc hydroxy carbonate
Polyethylene Glycol
Limonene
Benzotriazole

Amylase
Propylene Glycol
Sodium Acetate
Citronellol
Linalool
bis(N, N', N''-trimethyl-1,4,7-triazacyclononane)-trioxo-dimanganese (IV) di(hexafluorophosphate) monohydrate
Colorant
Colorant
Colorant
