# Supplementary figures and images for: Hygiene Efficacy of Short Cycles in Domestic Dishwashers
Source: Microorganisms. 2025 Jun 30;13(7):1542. doi: 10.3390/microorganisms13071542 (PMC12299907; doi:10.3390/microorganisms13071542)

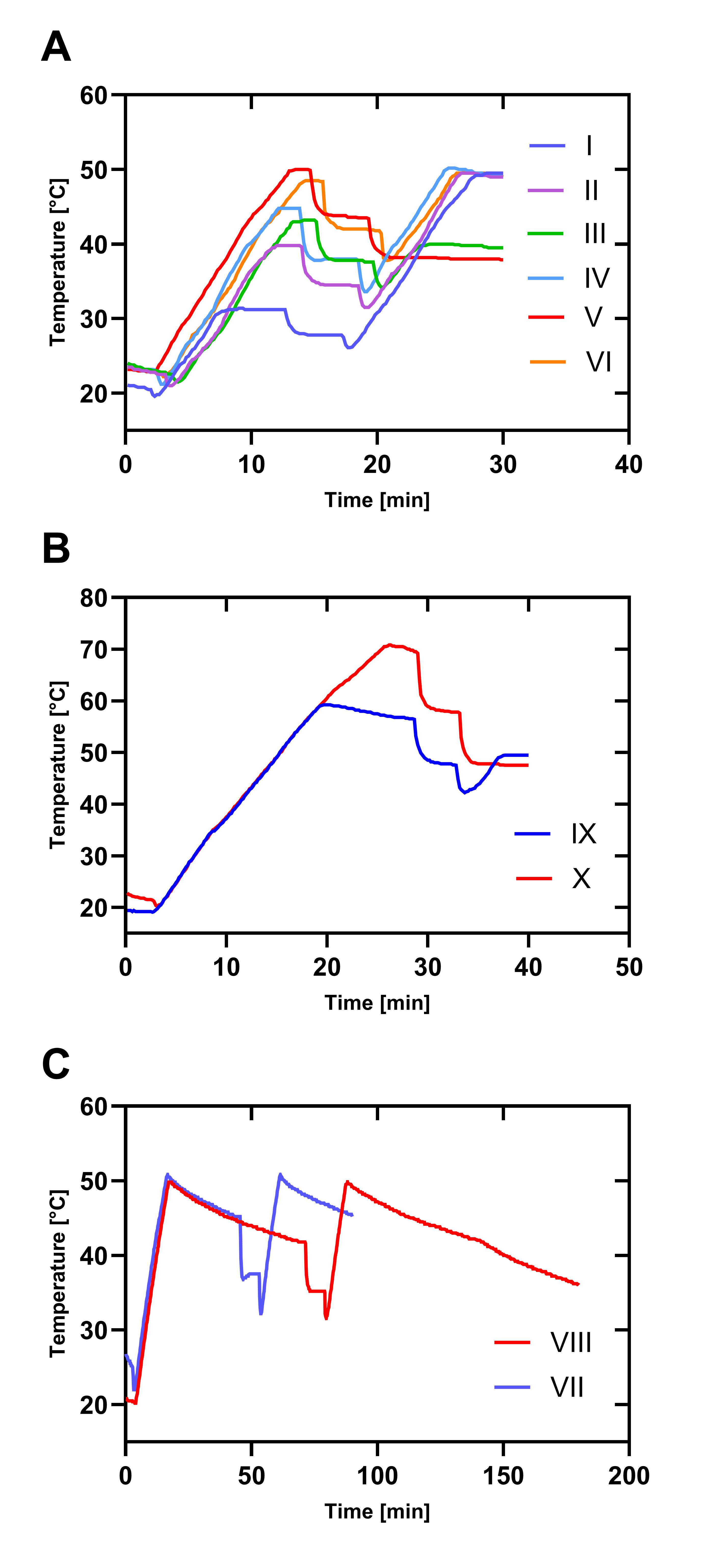

Supplement: Supplementary file 1 [file microorganisms-13-01542-s001.zip › microorganisms-3684142-supplementary/microorganisms-3684142 supplementary material/Figure S1.jpg]

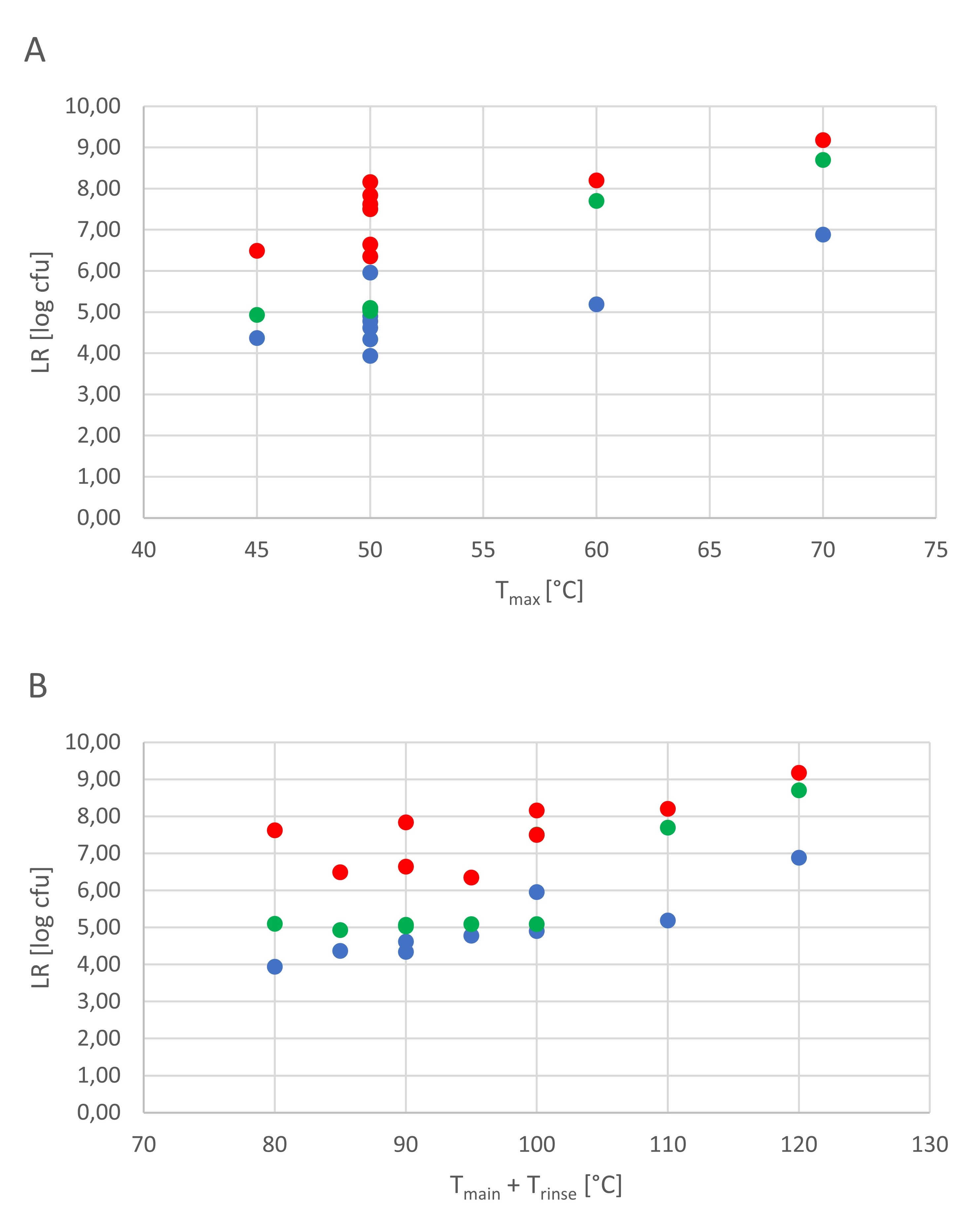

Supplement: Supplementary file 1 [file microorganisms-13-01542-s001.zip › microorganisms-3684142-supplementary/microorganisms-3684142 supplementary material/Figure S2.jpg]
